# The role of organizational and professional cultures in medication safety: a scoping review of the literature

**DOI:** 10.1093/intqhc/mzz111

**Published:** 2019-12-10

**Authors:** Samantha Machen, Yogini Jani, Simon Turner, Martin Marshall, Naomi J Fulop

**Affiliations:** 1 UCL Department of Applied Health Research, UK; 2 Centre for Medicines Optimisation Research and Education, UCLH NHS Foundation Trust, UCL School of Pharmacy, UK; 3 School of Management, University of Los Andes, Colombia; 4 UCL Research Department of Primary Care & Population Health, UK

**Keywords:** medication safety, medication errors, organizational culture, professional culture, safety culture

## Abstract

**Purpose:**

This scoping review explores what is known about the role of organizational and professional cultures in medication safety. The aim is to increase our understanding of ‘cultures’ within medication safety and provide an evidence base to shape governance arrangements.

**Data sources:**

Databases searched are ASSIA, CINAHL, EMBASE, HMIC, IPA, MEDLINE, PsycINFO and SCOPUS.

**Study selection:**

Inclusion criteria were original research and grey literature articles written in English and reporting the role of culture in medication safety on either organizational or professional levels, with a focus on nursing, medical and pharmacy professions. Articles were excluded if they did not conceptualize what was meant by ‘culture’ or its impact was not discussed.

**Data extraction:**

Data were extracted for the following characteristics: author(s), title, location, methods, medication safety focus, professional group and role of culture in medication safety.

**Results of data synthesis:**

A total of 1272 citations were reviewed, of which, 42 full-text articles were included in the synthesis. Four key themes were identified which influenced medication safety: professional identity, fear of litigation and punishment, hierarchy and pressure to conform to established culture. At times, the term ‘culture’ was used in a non-specific and arbitrary way, for example, as a metaphor for improving medication safety, but with little focus on what this meant in practice.

**Conclusions:**

Organizational and professional cultures influence aspects of medication safety. Understanding the role these cultures play can help shape both local governance arrangements and the development of interventions which take into account the impact of these aspects of culture.

## Introduction

Ensuring the safe use of medicines is a key priority of health systems worldwide, with an estimated annual global cost of US$42 billion [1]. Medication safety refers not only to the safe prescribing, dispensing and administration of medicines [2] but also to medication errors, defined as ‘preventable events that lead to actual harm’ [3]. Medication safety is a complex concept, with errors often having multifactorial origins across the different stages of the prescribing, dispensing and administration [3].

**Table 1:** Concepts included in review

Concept	Keywords
Medication safety	Medication errors, adverse events, medicines reconciliation, medicines optimization, reporting errors, near-misses
Culture	Organizational culture, professional culture, safety culture, shared values, shared beliefs, shared attitudes and shared behaviours
Professional groups	Nurses, nursing, doctors, physicians, medicine, pharmacists and pharmacy

**Table 2:** Working definitions of the two aspects of culture

Concept	Definition
Organizational culture	‘the pattern of shared basic assumptions-invented, discovered or developed by a given group as it learns to cope with its problems of external adaptation and internal integration … to (teach) new members as the correct way to perceive, think and feel in relation to those problems’ [8]
Professional culture	‘Values, beliefs, attitudes, customs and behaviours attached to a profession’ [81, 82]

**Figure 1:**
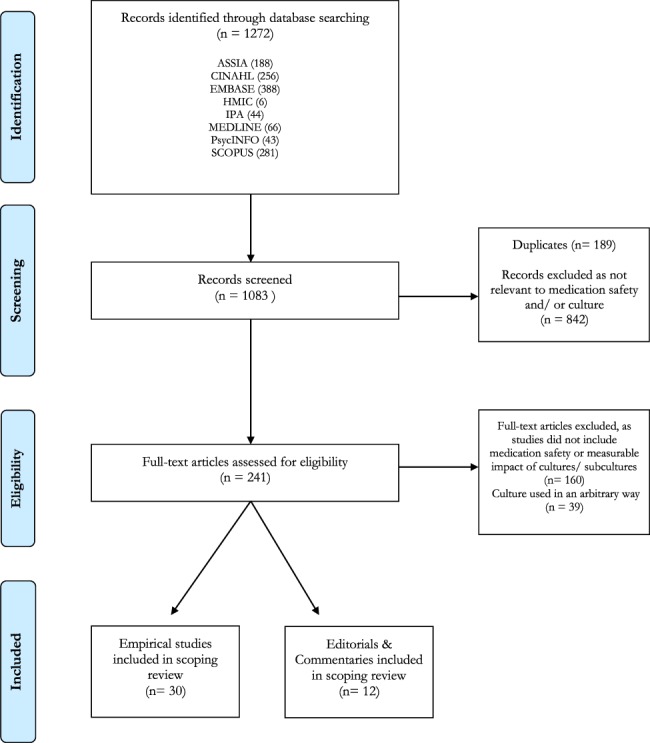
PRISMA(92) flow chart diagram of the scoping review process.

The contribution of organizational culture to health systems and its role in failures in care is well-known [4–7]. Organizational culture can be defined as ‘the way things are done around here,’ encompassing shared values, behaviours and attitudes among members in an organization, thus influencing the way day-to-day activities are carried out [8–10]. The role of organizational culture is discussed both in pharmacy and nursing research. In pharmacy practice research, organizational culture has been discussed in the context of patient safety, for example, in light of dispensing errors, but also wider within its role in implementing organizational change [11, 12]. Within the nursing literature, organizational culture is viewed in light of wider healthcare performance [13]—for example, in light of facilitating systems change [14] and nursing turnover and satisfaction [15]. In the medical literature, there is less of a focus on organizational culture’s role on both wider performance and patient safety more generally.

‘Sub-cultures’ within organizations may also exist, serving to reinforce or challenge wider organizational aims [10, 16–18]. It has been argued that professional sub-cultures can be among the ‘strongest’ cultures within an organization [19, 20]. It is suggested that a safety culture can only be sustained if there are a shared set of beliefs, attitudes and norms around what is ‘safe’ [21]. Therefore, culturally accepted norms attached to professions may impact medication safety [22, 23]. Research into incident reporting and operating theatre safety has identified the important role that cultures and sub-cultures can play [21, 24]. Currently, there is limited medication safety research identifying the role of these cultures. The aims of this scoping review were first, to explore the way in which culture was understood within medication safety—that is, how it is being defined and used within the context of medication safety research and second, to explore the role of professional and organizational cultures in medication safety.

## Methods

Arksey and O’Malley’s scoping review framework was used, which involves four steps: identifying the research question and relevant studies, selecting the studies, charting the information and reporting the results [25]. Scoping review methodologies have been used in recent years within the patient safety literature [26–28] and are useful in enabling a mapping of a broad field of research including different study designs and identifying gaps in the literature [25, 29]. Although later modifications of scoping review methodology have aimed to include a quality assessment stage [29–31], we did not include a quality assessment to ensure that relevant studies were included (e.g. insights into hospital reports of medication errors).

### Search strategy

The following databases were searched: The Cumulative Index to Nursing and Allied Health Literature (CINAHL), Medical Literature Analysis and Retrieval System Online (MEDLINE), EMBASE, SCOPUS, International Pharmaceutical Abstracts (IPA), Applied Social Sciences Index and Abstracts (ASSIA), PsycINFO, Health Management Information Consortium (HMIC) and the Cochrane Library. We searched these databases using key words attached to medication safety, culture and professional groups. Tables 1 and 2 highlight how these concepts were operationalized for the review. We focused on nurses, doctors and pharmacists as they have explicit roles within medication safety. An example search strategy can be found in the supplementary material.

### Study selection

Titles and abstracts were screened by SM based on the eligibility criteria available in the supplementary material. Studies were included if they clearly defined or conceptualized ‘culture’ and its role on any aspects of medication safety. To ensure reliability of the full-text review, 10% of the full-text articles were screened and reviewed by YJ. If agreement was >90%, then SM continued the screening process alone and at this stage agreement was 100%.

### Charting the data and synthesis of results

Data were extracted for the following characteristics: author(s), title, year of publication, study location, methods used, medication safety focus, professional groups, culture focus and role of culture upon medication safety focus. We utilized both integrative and interpretive methods [32, 33]. Integrative methods combine and pool data, whereas interpretive methods use induction and interpretation to synthesize different types of literature [32, 33]. Integrative methods were used to summarize findings from the included studies and interpretative methods were used based on an extensive iterative-inductive analysis framework [34]. Relationships within tabulated themes were explored; for example, comparing professional attitudes and norms across different aspects of medication safety, different professions and studies. Themes were reviewed by all authors.

**Table 3:** List of all included studies in review

Article	Country	Year	Medication safety focus	Role of culture
Aarts [70]	The Netherlands	2013	Prescribing	Prescribing is a social act which shows the authority of the doctor in being able to ‘solve’ the patient’s problem. Electronic prescribing challenges embedded social norms attached to professions. The author argues that interventions should take into account cultural nuances that may exist
Aboshaiqah [83]	Saudi Arabia	2013	Medication error reporting	Administrative response to error and fear was an important barrier to nurses in reporting medication administration errors. Staff believed there was too much emphasis placed upon errors as a marker of quality of care and errors weren’t looked upon from the perspective of the system-only the individual
Alqubaisi *et al.* [44]	United Arab Emirates	2016	Medication error reporting	Nurses and pharmacists’ likelihood to report errors was impacted by the perceived professional hierarchy and ‘power’ of doctors-with doctors wanting less formal platforms for errors to be discussed (i.e. a conversation). Pharmacists, doctors and nurses elicited a fear of reporting-worrying it would impact their reputation or result in litigation. Nurses elicited a greater fear of litigation
Andersen [84]	Denmark	2001	Prescribing	Professional culture as a barrier to collaborative working. The authors report that nurses and doctors had strong professional identities and when implementing a new drugs record system, the intervention challenged established roles of nurses and doctors. The authors characterized nurses by collectivism, whereas the doctors were more competitive and individualistic
Boyle *et al.* [55]	Canada	2011	Medication error reporting	Supportive organizational culture, defined as ‘enthusiasm about improved reporting … creating a safe reporting environment,’ was found to be an significant variable in ensuring reporting of medication safety incidents
Chiang *et al.* [85]	Taiwan	2010	Medication error reporting	Failure to report was strongly influenced by nurses’ experience of making medication administration errors. Barriers to reporting included fear, perception of nursing quality and perception of nursing professional development
Debono *et al.* [69]	Australia	2013	Compliance with protocols	There are deviations of medication safety practices across areas—for example, not checking identification bands in long term wards, as the staff are familiar with the patients
Dickinson *et al.* [68]	USA	2012	Safe Medicines Administration	Norms are passed on within professions and role modelling of these norms by professionals ensure they become common practice
Dolansky *et al.* [60]	USA	2013	Reducing medication errors/investigation into errors	Case study of a medication error. Looking at the error from an organizational and professional lens, the study identified that the unit level’s response to an error is important, as well as communication between nurses and senior staff
Federwisch *et al.* [86]	USA	2014	Interventions	This study, an evaluation of an intervention to reduce interruptions to nurses during their drug rounds, found that nurses elicited that the signs (to not interrupt) were not respected. The vests worn by nurses were poorly received, with the authors postulating that the ‘vests signify unavailability which didn’t mesh well with a nursing culture that values flexibility and availability’
Flanders and Clark [49]	USA	2010	Reducing medication errors/investigation into errors	Interventions aiming to limit interruptions to nurses during their drug rounds were fraught with difficulties because they implicitly challenged a nursing culture that pride themselves on availability and ability to multitask
Furmedge and Burrage [71]	UK	2012	Prescribing	Doctors arrived with limited knowledge of prescribing, however the authors argue that due to exposure to other colleagues within the profession, bad habits flourish which become difficult to ‘stamp out’
Gill *et al.* [66]	Australia	2012	Compliance with protocols	Medication related practices became routine practices by ‘the nursing gaze’—a term coined by the authors which was defined as a ‘surveillance strategy by which nurses examined each other’s activities.’ ‘Ward culture’ was important, where some areas were more diligent than others in complying with protocols. Junior nurses were discouraged from following protocols (e.g. checking ID bands) by their senior colleagues-with the authors concluding negative role modelling and active discouragement were key influences on practices being reinforced
Gimenes *et al.* [57]	Brazil	2016	Safe Medicines Administration	Trust and cooperation among staff in an intensive care unit influenced medication safety culture. On a wider organizational level, the trust had a zero tolerance to errors and the frequent firing strengthened a culture of punishment. There was a focus on the person making the error, rather than improving processes. On a professional level, nurses believed pharmacists didn’t understand their day-to-day difficulties and there was a lack of communication
Hammoudi *et al.* [58]	Saudi Arabia	2018	Medication error reporting	Medication errors were influenced by the communication process between nurses and doctors, and the reporting of these errors was influenced by nurses’ fear of reporting, and the administration response to the error—for example, a nurse being individually blamed for the error
Hartnell *et al.* [43]	Canada	2012	Medication error reporting	Professionals reported a fear that reporting an error would threaten their professional identity by appearing incompetent to colleagues. Organizational barriers, or ‘how things are done around here,’ resulted in an ineffective reporting system and no learning from errors
Hawkins *et al.* [63]	USA	2017	Systems evaluation	Pharmacists were an important ‘stop-gap’ between errors getting to the patient, however the authors reported that the pharmacists did not formally supporting the reporting of these errors. There were examples of interventions taken by the pharmacist to correct doctors’ errors but there were no examples of pharmacists informing the doctors of their error. This lead the authors to conclude that inter-professional hierarchies remain an issue within medication safety
Hemphill [56]	USA	2015	Interventions	The authors argue that while organizations may feel punitive responses to error are justified; it will not serve to be useful in understanding the processes and systems that contributed to the error. They claim that leadership is the ‘driving force’ behind a safety culture, where frontline staff will only believe safety is important if their senior leaders encourage this
Hong and Li [38]	China	2017	Medication error reporting	Safety climate was the most important domain impacting a unit’s safety culture. Nurses considered the unit’s overall safety culture as influential in reporting adverse events—where a flexible organizational culture promoted patient safety and reporting of adverse events due to instilling trust among the nurses
Hughes and Ortiz [52]	USA	2005	Reducing medication errors/investigation into errors	Clinicians have preconceptions about patient safety more generally which impacts their likelihood to engage in reporting and learning from errors. The authors comment that common feelings attached to reporting error are fear of being blamed, fear of litigation or serious impacts to the individual’s career
Hughes *et al.* [87]	UK	2007	Prescribing	Patterns of prescribing represent the visible artefacts of organization members but greater focus should be placed upon how these cultural patterns are created, communicated and maintained
Jacobs *et al.* [46]	Manchester, UK	2011	Medicines reconciliation/advanced pharmacy	There is a range of evidence describing aspects of organizational culture and its impact upon community pharmacy without explicitly defining this as organizational culture in this context. Authors identified five dimensions of organizational culture: the professional-business role dichotomy; workload, management style, social support and autonomy; professional culture; attitudes to change and innovation; and entrepreneurial orientation
Janmano *et al.* [62]	Thailand	2018	Medication error reporting	In a social network analysis of reporting medication errors, pharmacists were found to be central in the network of consultation on medication and were discussed to be ‘bridges’ between nurses and doctors
Kagan and Barnoy [35]	Israel	2013	Medication error reporting	The correlation between patient safety culture at an organizational level and error reporting rate was significant, leaving the authors to deduce that the ‘better’ the organizational culture for patient safety was, the more likely nurses were to report errors
Kavanagh [50]	Republic of Ireland	2017	Reducing medication errors/investigation into errors	Discusses the role of medication incidents on nurses-identifying that they feel guilt or too heavily bear the blame, leaving them to feel incompetent. There is a need for a blame free culture which can empower staff to report, with the authors arguing that this will have a significant impact on whether or not errors are noticed and reported
Kaissi *et al.* [64]	USA	2007	Reducing medication errors/investigation into errors	The use of practice guidelines was associated with reduced medication error rates in groups with a more ‘collegial’ culture-defined as a culture fostering coordination within doctors and between nurses and doctors
Kelly *et al.* [67]	USA	2016	Interventions	This article discusses the role of workarounds which can serve as a barrier to successful implementation of an intervention. These solutions, implemented by the nurses as solutions to their everyday issues, were found to be deeply ingrained into practice-so instead of being seen as workarounds or hazards to the staff, they are common and accepted practice
Liu *et al.* [61]	Australia	2011	Medication error reporting	Communication is influenced by hierarchy that exists in organizations and relates generally to vertical hierarchies, resulting in role conflict and struggles with interpersonal power and relationships
McBride-Henry and Foureur [88]	New Zealand	2006	Safe Medicines Administration	Staff nurses did not feel that senior leaders in their hospitals listened to their concerns and at times, nurses believed that they felt powerless to address the medication safety incidents they identified because it was beyond the scope of their position. Communication across the multi-disciplinary team was seen as important to nurses
Moody [48]	USA	2006	Medication error reporting	On the behavioural motivation scale, items involving worry, fear, anger and criticism relative to medication errors were scored highly by nurses. Nurses felt that the authority gradient in their organization hindered their ability to confidently engage and ask questions with someone of ‘higher’ authority. 2/3 of nurses described themselves as ‘prudent’ when dealing with authority and would only speak up if they deemed themselves to have ‘proper’ authority. Across different organizations, significant differences in nurses’ perceptions of their safety culture relative to different managers and supervisors
Ramsay *et al.* [22]	UK	2014	Compliance with protocols	Nurses were more engaged with medication score-card feedback than doctors, which identified contrasting approaches to improvement in the two professions. Nurses reported being more likely to be removed from their responsibilities if a drug error happened. Examples of ‘normalised deviance’ where errors were seen to be normal or unsurprising across areas and professions
Reid-Searl and Happell [59]	Australia	2011	Safe Medicines Administration	Participants of the study referred to the culture of the specific units and hospitals and claimed this had an important influence on the way they administered drugs and discussed the need to ‘fit in’ to the environment
Sahay *et al.* [53]	Australia	2015	Medication error reporting	Graduate nurses reported feeling reluctant to approach nursing colleagues for advice on medication administration. Graduate nurses experienced condescending language and felt nurses were impatient with their questions. Nurses also identified they felt blamed for medication errors that were made by doctors or other nurses
Salem *et al.* [51]	UK	2013	Medication error reporting	There was an organization-wide apathy to reporting as respondents elicited it was unlikely to respond with any meaningful feedback or change. Junior staff reported anxiety in challenging their more ‘superior’ colleagues. The authors identified that reporting errors was influenced by a feeling of protecting oneself and subsequent obligation. Respondents found that the response to an error by a nurse was more of an ‘open book,’ but when a doctor made a mistake it was ‘kept quiet’ with no learning of what happened as a result
Samsuri *et al.* [36]	Malaysia	2015	Medication error reporting	Pharmacists working in health clinics were 3.7 times more likely to have positive overall safety culture scores compared to pharmacists working in the hospitals. Reporting practices were impacted by the overall safety culture
Sarvadikar and Williams [45]	UK	2009	Medication error reporting	Nurses, doctors and pharmacists felt criticism and blame would follow if they reported an error. Nurses showed a greater fear of disciplinary action or even losing their jobs after an error compared to pharmacists and doctors. Nurses and pharmacists indicated they would report all errors irrespective of the patient outcome, whereas doctors were more likely to report the error if the patient’s condition worsened
Smetzer *et al.* [54]	USA	2003	Safe Medicines Administration	Hospitals that elicited a non-punitive approach to error reductions were more likely to be better at detecting, reporting and analysing errors. There were strong correlations between a strong supportive culture and the reporting of errors
Smits *et al.* [89]	The Netherlands	2012	Reducing medication errors/investigation into errors	In the same hospital, surgical and medical units were more likely to report errors compared to the emergency department. Non-punitive response to error, hospital management support and willingness to report were influenced by the overall safety culture in the units
Tricarico *et al.* [90]	Italy	2017	Medication error reporting	Incident reporting rates by profession were analysed prospectively and saw an increase of doctors engaging with reporting. The authors state that doctors started with initial scepticism before increasing involvement and reporting practices and that the incident reporting system was nor fully accepted by their own professional improvement ideologies
Turner *et al.* [23]	UK	2013	Medicines reconciliation/advanced pharmacy	Attitudes towards medication safety varied across professions-with pharmacists believing it was central to their hospital socialization. Attitudes were shaped by the social norms practices within the specialities and therefore awareness of medication safety practices differed across different specialties. Nurses elicited a higher likelihood to report errors due to their fear of litigation
Vogus and Sutcliffe [91]	USA	2001	Medication error reporting	Units within hospitals exhibit significant variation in safety organization and perceived trust in leadership. Unit-level leaders are able to enhance the effects of safety organizing on patient safety by fostering trust, thus making clinicians feel safe to discuss near-misses and errors
Wakefield *et al.* [37]	USA	2011	Medication error reporting	Explored role between four culture types, group-type culture, development-type culture, hierarchical-type culture and rational-type culture and reasons why medication administration errors were not reported. A hierarchical or rational-type culture was negatively associated with reporting of medication administration errors

## Results

A total of 42 articles were selected for review after removal and duplicates and screening (Fig. 1), with 30 empirical studies and 12 pieces of grey literature. These articles are summarized in Table 3. Study characteristics can be seen in Tables 4–6. In studies looking at professions, the main focus was on solely nursing staff (*n* = 19), nurses, doctors and pharmacists (*n* = 10) and nurses and doctors (*n* = 6). Of the 30 empirical studies, 17 utilized quantitative methods, 10 qualitative methods and 3 mixed-methods.

### Conceptualization of culture

We aimed to explore the way in which culture was conceptualized in medication safety, on both organizational and professional levels. Of the 241 full text articles assessed, 39 were excluded as they were judged to use the term ‘culture’ in an unfocused and arbitrary way. In some cases, articles cited improving culture as a means of improving medication safety but without defining or conceptualizing culture. Excluded articles also discussed improving culture as a mediator in achieving organizational or improvement aims, yet offered little on what this meant in practice.

In studies focusing on organizational culture, the majority of articles utilized a tool to measure organizational culture, for example, the Stanford/Patient Safety Centre of Inquiry culture survey [35], the Safety Attitudes Questionnaire [36] and the Culture Inventory [37]. At times, unit level culture was used interchangeably with safety culture, a just and a blame-free culture. In the studies focusing on professional cultures, only one utilized a tool (the Patient Safety Culture Assessment Scale [38]), with the remaining articles referring to shared values, attitudes, norms, behaviours or views among and within professions, which matched our definition of professional culture. ‘Professional culture’ as a phrase was used less often in the articles than ‘organizational culture.’

**Table 4:** Included articles by culture focus

Culture focus	Included in analysis (%)
Organizational culture	11 (26)
Organizational culture and professional culture	14 (44)
Professional culture	17 (40)

**Table 5:** Included articles by medication safety focus

Medication safety focus	Included in analysis (%)
Compliance with protocols	3 (7.14)
Interventions	3 (7.14)
Medication error reporting	18 (42.86)
Medicines reconciliation/advanced pharmacy	2 (4.76)
Prescribing safety	4 (9.52)
Reducing medication errors/investigation into errors	6 (14.29)
Safe medication administration	5 (11.9)
Systems evaluation	1 (2.38)

**Table 6:** Included articles by country/region

Country/region	Included in analysis (%)	Country/region	Included in analysis (%)
Australia	5 (11.9)	Republic of Ireland	1 (2.38)
Brazil	1 (2.38)	Saudi Arabia	2 (4.76)
Canada	2 (4.76)	Taiwan	1 (2.38)
China	1 (2.38)	Thailand	1 (2.38)
Denmark	1 (2.38)	The Netherlands	2 (4.76)
Israel	1 (2.38)	The UK	7 (16.67)
Italy	1 (2.38)	The USA	13 (30.95)
Malaysia	1 (2.38)	UAE	1 (2.38)
New Zealand	1 (2.38)		

### Themes

**Figure 2:**
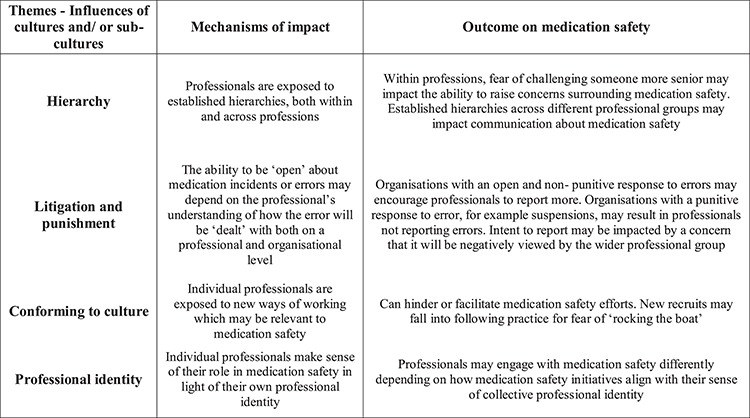
Themes from the review.

Four themes emerged from the data synthesis, describing both the direct and indirect impact of organizational and professional culture on medication safety practices. Three of the four themes were cross-cutting across professional and organizational levels, alluding to the difficulties in disentangling professional and organizational cultures, as individuals and communities may be a function of both their profession and their organizational setting. A visual depiction of these themes can be found in Fig. 2.

#### Theme 1: professional identity

This theme focuses on the way professionals make sense of their role in medication safety relative to their professional identity. Professional identity is defined as ‘the various meanings attached to oneself by self and others’ [39] and it is an important cognitive mechanism impacting professionals’ attitudes, behaviour, values and beliefs in the work place [40–42]. For pharmacists, medication safety was inherent in their role and resulted in a belief that it was their place to ‘champion’ medication safety [23]. Nurses shared the view that medication safety was important and maintained through their professional role [43], which resulted in engagement with reporting structures [44], aligning with a profession already engaged in incident reporting. Doctors were less likely to see medication safety as part of their professional role relative to other clinical skills [23] and less likely to report medication errors as they believed nurses were more likely to do so for them [44]. Nurses and pharmacists were more likely to report an error irrespective of the level of patient harm, whereas doctors were more likely to report if patient outcomes worsened due to the error [45]. Fear of tainting their professional reputation by reporting an error was discussed by nurses, doctors and pharmacists [44, 46–50].

#### Theme 2: litigation and punishment

This theme relates to the professional and organizational response to medication errors. On a professional level, fear of litigation and punishment due to medication error was identified by nurses, pharmacists and doctors [23, 47, 48, 51, 52]. For nurses, this resulted in increased reporting of errors and compliance to documentation in order to protect themselves [23, 51]. Nurses believed they were more likely to be held accountable for errors compared with doctors [45, 48, 53]. Organizations fostering a non-punitive response to errors were said to result in better detection, reporting and analysing of medication errors [54–56]. Hospitals with a punitive response to error, cited as having a ‘blame culture,’ resulted in poor reporting and learning across the organization [47, 55, 57]. In one case, the ‘frequent firing’ of staff committing serious drug errors strengthened a culture of fear and punishment [57]. Professionals elicited a concern that medication safety reports were not used ‘correctly’ by their organization, for example, not to drive improvement but as a measure of the quality of nursing care [58].

#### Theme 3: hierarchy

The concept of professional hierarchy operated both within and across the three professions. Hierarchy within the nursing and medical profession negatively impacted nurses’ and doctors’ intention to report errors [37, 51, 59, 60]. Junior doctors saw their perceived lack of experience and knowledge as a barrier to seeking support or challenging senior colleagues, thus inhibiting them from reporting errors and near-misses [51]. Newly qualified nurses felt intimidated by senior nurses due to their grading or experience [60], and felt obliged to fall in line with existing, and at times unsafe, practices for fear of ‘rocking the boat’ [59]. On an inter-professional level, nurses and pharmacists argued that perceived importance or ‘power’ of doctors inhibited reporting doctors’ medication errors or near-misses [44, 48, 61]. One article identified that nurses saw themselves as ‘prudent’ when dealing with authority—only challenging another professional if they had the ‘proper authority’ [48], however what constituted ‘proper authority’ was not explored. Interruptions to nurses’ drug rounds were accepted based on hierarchical organizational relationships—for example, the nurse’s perceived ‘power’ of the person wanting their attention [49]. Pharmacists were considered central figures within medication safety, supporting positive interactions across the professional groups and acting as ‘bridges’ between professional groups [62]. However, one study reported that while pharmacists were an important ‘stop-gap’ between errors getting to the patient, doctors were not informed of their errors leaving the author to conclude inter-professional hierarchies were a barrier to learning from errors [63]. Areas with a more ‘collegial culture,’ defined by increased coordination and communication between physicians and nurses, were statistically more likely to engage in medication safety practice guidelines and report lower medications error rates [37, 64].

#### Theme 4: conforming to culture

This theme relates to the role of enculturation of individuals into medication safety practices. Enculturation refers to the process of an individual’s socialization into the accepted norms, practices, attitudes and values of the group [65]. New recruits discussed a pressure to conform to the expected and established norms in an area or within their professional group. The priority given to medication safety was influenced by social networks and the attitudes and practices within their professional group or organizational setting [63]. The enculturation of new recruits was well discussed within the nursing profession and could either serve to facilitate or hinder medication safety [53, 59, 66–68]. Some wards were cited as having a ‘better’ safety culture—facilitating the reporting of errors [35] and here were examples of medication safety practices (e.g. second checking intra-venous drugs) varying significantly across different wards within the same hospital [66, 69, 70]. Junior or inexperienced nurses felt pressured to ‘fit in’ with the established practices as their colleagues were impatient with the time it took them to complete a drug round, which in some cases resulted in the administration of drugs they knew to be incorrect [53, 60]. These embedded and established practices were communicated explicitly and implicitly to new members of nursing staff [66]. One study coined this implicit communication as the ‘nursing gaze,’ where medication-related practices became routine as nurses surveyed each other’s activities and practices [66]. Within medicine, newly qualified doctors started out with little knowledge of prescribing and picked up ‘bad habits’ as they were exposed to prevailing informal practices [71].

## Discussion

We set out to identify how the concept of ‘culture’ has been used in the medication safety literature. 16% of the articles screened for inclusion used ‘culture’ in an arbitrary way—that is, used rhetorically without being critically explored. While the increased focus on ‘culture’ in health care has facilitated a better understanding of how health care organizations work, it has been argued at times that culture as a concept has been poorly conceptualized or used superficially as a panacea for improvement [72]. This inadequate conceptualization is not only due to culture being used in a remedial sense, but also due to the complex and conflicting schools of thought of what culture is and how it can be conceptualized in health care. Defining culture has well-established difficulties [73, 74], famously demonstrated by anthropologists Krober and Klukholm, who sought to review different concepts and definitions of culture and assembled 164 different definitions from this research [75].

What culture ‘is’ is also a well-established debate. Organizational culture has been studied in a dual way, as either a root metaphor or variable. Culture as variable identifies a positivist view where culture is something an organization *has*, where specific cultural aspects may be isolated or manipulated in light of organizational aims [73]. Culture as a root metaphor identifies culture as something the organization *is* and therefore not readily identifiable or separate to the organization. As culture is a function of the specific context and subtle social and historical processes in the organization [76, 77], it may offer less power for managerial control [78]. In the wider patient safety literature, the role of culture is presented in two different ways: as a ‘culprit’ for poor outcomes and also as a remedy for improving outcomes [5]. The arbitrary use of the term ‘culture’ in some of the reviewed articles alluded to this positivist and remedial view of culture as a variable, which can be manipulated or ‘improved,’ to achieve medication safety aims. There is a continuing rhetoric and normative desire within health care to move from a toxic or ineffective culture towards a positive, supportive and open culture. While this overarching aim is undeniably important, at times it results in research which incorporates this message, but with little understanding of the depth or sophistication of enquiry needed to support such a message.

We set out to explore the impact of professional and organizational culture on medication safety. Established aspects of professional organization, for example, a profession’s hierarchy, was identified as having an impact on medication safety practices. During this review, collective attitudes and norms held within professions and towards other professional groups (e.g. others´ perceived ‘power’) featured heavily in the papers included. As medication safety involves collaboration between nurses, doctors and pharmacists, comparing collective cultural views expressed by professionals with their actual daily practice would aid understanding of how power affects collaboration on medication safety (e.g. by triangulating interviews and observational evidence). This particular finding echoes a recent inquiry into the prescribing practices at Gosport War Memorial Hospital in England, where inappropriate and unsafe opioid prescribing by doctors went largely unchallenged by nurses and pharmacists [79]. The way professionals make sense of their role and others’ roles is important in understanding engagement with medication safety and subsequent initiatives. This corroborates a study of reporting practices among doctors, where they believed that ‘form filling’ and ‘paper work’ associated with reporting was more appropriate for professions suited to bureaucratic procedures and more amenable to managerial control—for example, nurses [24]. Finally, the role of enculturation meant that new recruits were socialized into accepted norms which, at times, resulted in unsafe practices. This illuminates the role of professional communities in passing on norms explicitly and implicitly which supports previous research identifying informal communication between processionals as a key model of learning within health care, with such models important in organizational learning [80].

To the authors’ knowledge, this is the first review that has explored the role of organizational and professional cultures within medication safety. Individual articles included in this review focused on one aspect of medication safety or from the perspective of one professional group; and by synthesizing findings from different studies, the role of shared norms, attitudes and practices in professional groups upon medication safety can be better understood. De Bono has previously suggested that professional sub-cultures are ‘often stronger than other groupings within an organisation’ [19, 20] and this review helps to explore the role of professional cultures. If we see organizations, for example, hospitals, as being made up of different sub-cultures, then this review enables an understanding of why interventions must take these sub-cultures into account. This raises the importance of ensuring managers are engaged with understanding the nuances in collective views, values or ‘cultures’ that exist in professional groups and organizational settings and how these may inform, and influence, governance of medication safety.

## Limitations

Due to aforementioned complexities, conducting a sensitive and precise search strategy was challenging. This review compared reports of ‘culture’ across different health care settings and as professional practice may vary significantly across countries and organizations, comparisons of professional cultures may be best served to be conducted in the same country and/or organization. This review has surfaced the intricate link between organizational and professional cultures and as these two concepts are not mutually exclusive, this added to the challenges in conducting a review on such a nebulous concept. Recent debates in scoping review methodology include arguments for discussing findings with relevant stakeholders, originally an optional step in Arksey and O’Malley’s framework, which would add insight by bringing actors’ experiences to bear on provisional findings [30]. This review did not include such feedback on the findings, and therefore this is a limitation. Finally, 45% of included articles focused solely on the nursing profession and therefore, future research should endeavour to study nurses, doctors and pharmacists within the same organizational setting (both wards and hospitals) to explore the role of sub-cultures upon medication safety practices.

## Conclusion

Our scoping review provides a wider understanding of the importance and role of cultures in medication safety. Differences in perception of professional roles acted both as a barrier and facilitator to medication safety. Inter and intra-professional hierarchies influenced the communication within and across professional groups, with some cases professionals hesitant to question medication orders for fear of challenging their superiors. Implicit and explicit socialization into culturally accepted norms, both across an organization setting and a profession, was important in embedding medication safety practices. Medication safety is high on the current policy agenda and understanding the role of organizational and professional cultures allows for interventions and governance strategies to be shaped in light of the impact of these important concepts.

## Supplementary material

Supplementary material is available at *INTQHC Journal* online.

## Funding

Health Foundation Improvement Science PhD Fellowship and supported by the National Institute for Health Research (NIHR) Collaboration for Leadership in Applied Health Research and Care North Thames at Barts Health NHS Trust. The views expressed are those of the author(s) and not necessarily those of the NHS, the NIHR or the Department of Health and Social Care. YY is an NIHR Senior Investigator.

## Supplementary Material

Supplementary_mzz111
