# Supplementary material for: The role of organizational and professional cultures in medication safety: a scoping review of the literature
Source: Int J Qual Health Care. 2019 Dec 10;31(10):G146–57. doi: 10.1093/intqhc/mzz111 (PMC7097989; doi:10.1093/intqhc/mzz111)
Supplement: Supplementary_mzz111 [file supplementary_mzz111.docx]

**Supplementary material**

Example search strategy:

| **Theme/ Concept** | **Search Terms** |
| --- | --- |
| Medication safety | Medication errors OR medication reconciliation OR safety adj3 (medicin* or medication*) OR error* adj3 (medicin* or medication*) OR patient* adj3 (harm* or safety) OR medicines optimi?ation OR adverse* adj2 events OR drug related problems |
| Culture (Professional Culture & Organisational Culture) | Organisational culture OR organi?ation* adj3 culture* OR professional culture* OR corporate culture* OR shared values OR social values OR social beliefs OR safety culture |
| Professional Groups | Exp Medical Staff, Hospital OR Exp Physicians/ OR Pharmacists/ OR nursing staff, Hospital/ or (Clinician* or doctor* or physician*) OR (nurs* adj3 hospital*) OR pharmacist OR professional groups |

**Inclusion and exclusion criteria for empirical studies**

|  | **Inclusion Criteria** | **Exclusion Criteria** |
| --- | --- | --- |
| Study Participants | - Any registered health care professional who is involved within the prescribing, administration or auditing of medications (e.g. nurses, physicians and pharmacists) - Residing in a country with a westernised health care setting (EEA, Central/Eastern Europe post-2004, Israel, N. America, Australasia, Singapore, Taiwan, Chinese SARs). - Studies reported in the hospital setting (e.g. in-patient ward) or community setting, such as a care home | - Health care professionals who are not directly involved in the prescribing, administration or auditing of medications - Residing in a developing nation, or nation without adequate universal health coverage and low HDI. - Studies reporting patient’s perceptions of medication safety (e.g. safety of self-administration of medicines in the hospital or community setting) |
| Intervention or Study Focus | - Any intervention targeted towards the improvement of medication safety where medication safety includes: a) medication reconciliation b) medicines optimisation c) efforts to reduce medication errors and adverse events d) efforts to increase the reporting of near misses and errors - Any primary research discussing barriers or facilitators to improving medication safety as defined above | - Any intervention or reporting not focused on improving medication safety e.g. another aspect of patient safety |
| Outcome Measurement | - Empirical research identifying the impact of professional or organisational culture upon medication safety practices e.g. as barriers or facilitators to above interventions - Organisational culture defined as “the pattern of shared basic assumptions- invented, discovered or developed by a given group as it learns to cope with its problems of external adaptation and internal integration…to (teach) new members as the correct way to perceive, think and feel in relation to those problems” - Professional cultures defined as “the culture of a profession is manifested in its members by a sense of community and by the bonds of a common identity”. - N.b. ‘Professional cultures’ as a concept does not necessarily have to be mentioned in the research if the role of attitudes, norms, values and beliefs are discussed in relation to relevant interventions identified above - Organisational culture to include many levels of organisations, from the role of board level culture upon interventions down the ward level culture impact. | - Uses of culture as an arbitrary word, where it is not defined nor its impact upon relevant interventions not quantified or qualified e.g. included in introduction or title - Studies reporting the impact of organisational climate upon medication safety interventions |
| Study Design | - Observational/epidemiological studies conducted in a real-world setting to demonstrate measurable impact of professional and/or organisational cultures upon medication safety practices - Measurable impact of professional and/ or organisational cultures may be implicit or explicitly stated | - Non- empirical studies - Commentaries, reviews and other grey-literature |
| Restrictions | - English language - Date of publication: Database inception- 2017 | - Abstract not in English. |

**Inclusion and exclusion for non- empirical studies (grey literature)**

|  | **Inclusion Criteria** | **Exclusion Criteria** |
| --- | --- | --- |
| Study Participants | - Any registered health care professional who is involved within the prescribing, administration or auditing of medications (e.g. nurses, physicians and pharmacists) - Health care (and non-healthcare) professionals who are not directly involved in the prescribing, administration or auditing of medications but involved in some capacity (e.g. clinical risk managers / senior clinical managers) | - Health care professionals who are not directly involved in the prescribing, administration or auditing of medications - Residing in a developing nation, or nation without adequate universal health coverage and low HDI. - Studies reporting patient’s perceptions of medication safety (e.g. safety of self-administration of medicines in the hospital or community setting) |
| Intervention or Study Focus | - Any intervention targeted towards the improvement of medication safety where medication safety includes: a) medication reconciliation b) medicines optimisation c) efforts to reduce medication errors and adverse events d) efforts to increase the reporting of near misses and errors - Discussing barriers or facilitators to improving medication safety as defined above | - Any intervention or reporting not focused on improving medication safety e.g. another aspect of patient safety |
| Outcome Measurement | - Research discussing the impact of professional or organisational culture upon medication safety practices e.g. as barriers or facilitators to above interventions - Organisational culture defined as “the pattern of shared basic assumptions- invented, discovered or developed by a given group as it learns to cope with its problems of external adaptation and internal integration…to (teach) new members as the correct way to perceive, think and feel in relation to those problems” - Professional cultures defined as “the culture of a profession is manifested in its members by a sense of community and by the bonds of a common identity”. - N.b. ‘Professional cultures’ as a concept does not necessarily have to be mentioned in the research if the role of attitudes, norms, values and beliefs are discussed in relation to relevant interventions identified above - Organisational culture to include many levels of organisations, from the role of board level culture upon interventions down the ward level culture impact. | - Uses of culture as an arbitrary word, where it is not defined nor its impact upon relevant interventions not quantified or qualified e.g. included in introduction or title - Studies reporting the impact of organisational climate upon medication safety interventions - Research identifying measurable impact of professional and/or organisational cultures |
| **S**tudy Design | - Data sources: Grey Literature, Reviews & Commentaries | - Empirical studies |
| Restrictions | - English language - Date of publication: Database inception- 2017 | - Abstract not in English. |
